# A False Dichotomy: RCTs and Their Contributions to Evidence-Based Public Health

**DOI:** 10.9745/GHSP-D-14-00245

**Published:** 2015-03-02

**Authors:** Laurel E Hatt, Minki Chatterji, Leslie Miles, Alison B Comfort, Benjamin W Bellows, Francis O Okello

**Affiliations:** a Abt Associates Inc., International Health Division, Washington, DC, USA; b Population Council, Lusaka, Zambia

## Abstract

Global public health should rely on those research methods that best answer the pressing questions at hand. Randomized controlled trials (RCTs) and other rigorous impact evaluation methods have a critical role to play in public health.

***See*** ***Shelton's response*** ***as well as related article by******Shelton*****.**

Editor-in-Chief James Shelton's editorial on September 12, 2014, concluded with a laudable call for studies in global health that increase understanding of the “‘how and when’ of implementation at scale”^1^^, p. 257^:

Bringing together evidence arising from different methodologies with sufficient detail to illuminate causal relationships is essential to applying such knowledge to real-world public health problems across diverse situations.

Indeed, triangulating among different information sources and reviewing evidence systematically are the hallmarks of nuanced, ethically conducted, effective public health research. However, we take issue with Shelton's opening statement that “randomized controlled trials (RCTs) have limited utility for public health” (p. 253). Rigorously designed randomized experiments and other impact evaluation methods have a critical role to play in global public health—not just for answering small-scale questions of efficacy but also for gauging the effectiveness of scaled-up approaches.

Setting RCTs in opposition to other systematic approaches for generating knowledge creates a false dichotomy, and it distracts from the more important question that Shelton addresses—namely, which research method is best suited for the question at hand? The choice of a research method is not an either/or proposition but depends on the particular research question to be answered and the context. So, when do RCTs make sense, what value do they add, and how do they relate to other research methodologies?

Setting RCTs in opposition to other systematic research approaches creates a false dichotomy.

Laying out a “theory of change” is a critical first step in designing any type of intervention or evaluative research, whether experimental or not; we agree strongly with Shelton on this point. Positing the causal pathway through which an intervention operates should frame *all* high-quality research. However, we view this as a prerequisite, not a substitute, for rigorous testing. Perhaps most fundamentally, it is still incumbent on global public health practitioners to answer definitively the question, “Does it work?” *before* asking, “How can it be made to work practicably at scale?” Programs that are assumed to work are sometimes shown—by random assignment—*not* to work; assumptions made based on common sense may turn out to be incorrect.

It is imperative for public health practitioners to answer definitively, “Does it work?” before asking, “How can it be made to work practicably at scale?”

Rigorous experimental studies continue to generate some of the highest quality evidence for demonstrating the efficacy and effectiveness of interventions, and they continue to demonstrate that expert intuition, although important, is insufficient. Here is one example. Economists have theorized that charging small fees for public health goods (such as insecticide-treated nets, deworming medicine, or water purification solution) could promote use and reduce wastage, thus increasing the efficiency of public subsidies.^2^^,^^3^ The logic is that the price would signal to consumers that a product had greater value than a free product, thus encouraging uptake, and also that the “sunk-cost” effect (“*I'd better use it since I already paid for it”*) would promote use. But a series of RCTs has shown that these assumptions are often incorrect. Charging a nominal fee for such health products substantially lowered uptake, especially among the poor,^4^^,^^5^ and distributing them for free did not make them more likely to be wasted by recipients.^4^^,^^6^^,^^7^

Once we determine “whether it works,” RCTs can also help assess whether a public health intervention is effective on a larger scale to inform roll out of health policy. In Rwanda, the phased, randomized roll out of a national performance-based payment scheme for primary health care providers (based on pilot experiences) enabled the government to demonstrate conclusively that this approach improved certain key utilization and quality indicators *at scale*.^8^ A randomized study on deworming school children in Kenya is an especially compelling example of policy makers using RCT results to inform broad policy changes. In this case, mass-treating school-aged children with deworming drugs every 6 months was found to reduce school absenteeism by one-quarter, at a very low cost per additional year of school participation.^9^ These findings ultimately led to several far-reaching initiatives: the Kenyan government launched a program to treat 5 million school-aged children annually; the State Government of Bihar in India implemented a mass deworming program; and the World Food Programme included deworming treatment in all of its school feeding programs.^10^

The best experimental research will also integrate qualitative, observational, and administrative data sources into the overall analysis. A rigorous mixed-methods approach is essential to untangle the *why* and *how* of the impact identified in an RCT—whether positive, negative, or absent. (As an aside, Shelton's editorial criticizes the World Health Organization's reliance on the GRADE system for the emphasis placed on RCTs in developing guidelines for public health, but the GRADE guidance itself notes the importance of observational studies and qualitative evidence.^11^) The evaluation of the PROGRESA conditional cash transfer program in Mexico illustrates the value of a mixed-methods approach. An experimental research design allowed researchers to establish that conditional cash transfers can improve child health, reduce anemia, and increase children's height.^12^ But program implementation benefited from other types of research and analysis: household surveys from the study sample,^12^ data from administrative sources, community questionnaires, school and clinic surveys, semi-structured interviews with stakeholders, and focus group discussions. This multipronged approach allowed researchers and policy makers to identify clearly *how* to refine and improve various structural and operational components of the complex program.^13^ Since then, similar programs have been tested and implemented in Brazil, Honduras, and Nicaragua.^13^

Mixed-method approaches are essential to untangle the *why* and *how* of the impact identified in an RCT.

RCTs do have well-documented limitations, some of which are outlined in Shelton's editorial, and conducting a randomized experiment may not always be possible even when it might be ideal. But one commonly made assertion is important to counter here—that RCTs have particularly burdensome cost requirements. Based on our experience, RCTs can be conducted for the same budget (or less) than pre-post or quasi-experimental design studies. In many instances, a baseline survey is not needed when interventions are allocated by random assignment. Furthermore, data analysis for RCTs is often more straightforward and therefore less expensive.^14^ Several of us recently implemented an evaluation, using face-to-face provider interviewers and mystery client surveys, of randomized assignment of drug sellers in Ghana to a text-messaging intervention; because an entire round of data collection was unnecessary, costs were substantially reduced.^15^

As with those who work on education, poverty alleviation, and other social-sector programs in developing countries (who frequently rely on evidence from randomized evaluations),^16^^,^^17^ global public health practitioners should use the most rigorous, systematic approaches available to answer questions and make decisions in the face of daily uncertainty. We can do far better than “intuition, trial and error, and experience.”^1^^, p. 255^

Shelton uses a compelling metaphor, arguing that using RCTs to design and implement public health programs at scale is like trying to light up a football stadium with a laser pointer. But we would counter that no single type of study can “illuminate the stadium.” We agree that RCTs cannot answer all public health questions, or light up the stadium alone, but neither can any other single method. It takes a combination of complementary research approaches to light up our field of public health, and RCTs have been shown to be a powerful and luminous force in that effort.
